# Epigenetic–Mitochondrial–Metabolic Crosstalk in Retinal Pigment Epithelium (RPE) Dysfunction in Age-Related Macular Degeneration (AMD)

**DOI:** 10.3390/antiox15060713

**Published:** 2026-06-04

**Authors:** Yijing Yang, Ying Deng, Xiang Li, Pai Zhou, Qinghua Peng, J. Arjuna Ratnayaka

**Affiliations:** 1 School of Traditional Chinese Medicine, Hunan University of Chinese Medicine, Changsha 410208, China; 004761@hnucm.edu.cn (Y.Y.); 004762@hnucm.edu.cn (Y.D.); 313322@hnucm.edu.cn (X.L.); zhoupai2020@stu.hnucm.edu.cn (P.Z.); 2 School of Clinical and Experimental Sciences (CES), Faculty of Medicine, University of Southampton, MP 806, Tremona Road, Southampton SO16 6YD, UK; 3 Institute of Ophthalmology and Otolaryngology of Chinese Medicine, Changsha 410208, China; 4 Changsha Centre for Innovation in Traditional Chinese Medicine Techniques for the Prevention and Treatment of Retinal Diseases and Visual Function Protection, Changsha 410208, China

**Keywords:** age-related macular degeneration, retinal pigment epithelium, epigenetic regulation, mitochondrial dysfunction, redox signaling, chromatin remodeling

## Abstract

Age-related macular degeneration (AMD) is a leading cause of irreversible vision loss in older adults and is characterized by progressive dysfunction of the retinal pigment epithelium (RPE). Although genetic susceptibility and environmental exposure both contribute to disease risk, the mechanisms through which chronic metabolic and oxidative stress are integrated into sustained RPE dysfunction remain incompletely understood. Increasing evidence from human AMD donor tissue and experimental RPE models indicates that epigenetic regulation operates at the interface between mitochondrial dysfunction, redox imbalance, and transcriptional remodeling. This review synthesizes current findings on DNA methylation, chromatin accessibility, histone modification, and RNA-based regulation in AMD, with emphasis on their metabolic and mitochondrial context. Studies in human AMD-RPE demonstrate that epigenetic alterations are generally selective rather than global and frequently involve pathways related to mitochondrial maintenance, lipid metabolism, oxidative stress responses, and cellular homeostasis. Mechanistically, mitochondrial dysfunction and reactive oxygen species (ROS) may influence epigenetic regulation through altered Nicotinamide adenine dinucleotide (NAD^+^) availability, acetyl-CoA metabolism, redox-sensitive chromatin regulation, and modulation of DNA methyltransferase and histone deacetylase activity. Redox-sensitive pathways, including antioxidant signaling, further connect mitochondrial stress to adaptive or maladaptive transcriptional responses in the RPE. Importantly, while several interactions discussed are supported by findings in human AMD tissue, other components of the proposed epigenetic–mitochondrial–redox framework remain inferential or model-based and require further validation. Rather than acting as isolated disease triggers, epigenetic changes are more likely to function as stress-responsive regulatory layers that stabilize transcriptional states over time in a long-lived post-mitotic tissue. We further discuss unresolved questions regarding causality, reversibility, therapeutic feasibility, and stage-specific intervention strategies. Collectively, this framework positions the epigenetic–mitochondrial–redox axis as a unifying model for understanding RPE vulnerability and AMD progression.

## 1. Introduction

Age-related macular degeneration (AMD) remains the leading cause of irreversible central vision loss in older adults, with a growing impact as populations continue to age. Analyses from the Global Burden of Disease project indicate that this increase is driven primarily by demographic changes rather than by abrupt shifts in individual risk profiles [1]. Estimates of the global AMD prevalence reinforce this view, which projects growth from approximately 196 million affected individuals in 2020 to nearly 288 million cases by 2040 [2]. This indicates that even in the absence of any major changes in the underlying disease mechanisms, healthcare systems will face mounting pressures as the number of older individuals with high risks of AMD steadily increases.

AMD does not develop through a single biological route. Instead, it emerges from the combined effects of aging, inherited susceptibility, and long-term environmental stress. Integrative studies that link genetic findings with disease biology show that risk variants converge on pathways involved in complement regulation, lipid metabolism, extracellular matrix remodeling, angiogenesis, and immune signaling [3,4]. Large genome-wide association studies support this framework while also revealing that many risk signals reside in non-coding regions, where regulatory control is as relevant as the DNA sequence itself [5,6]. This complexity is mirrored in clinical practice: individuals with broadly similar risk profiles often follow markedly different disease trajectories, implying that gene regulation and cellular plasticity shape how genetic risks are translated into diverse phenotypes.

Epigenetic regulation provides one route for linking these layers of influence. Epigenetic marks modulate gene expression without altering the DNA sequence and remain sensitive to metabolic state, oxidative stress, and inflammatory signals that can persist over long periods. Previous studies have described how DNA methylation, histone modification, and chromatin organization may contribute to both retinal development and disease, while also emphasizing that the retina follows regulatory principles that do not always mirror those of proliferative tissues [7]. Work focused on ophthalmic epigenetics has therefore highlighted the importance of these mechanisms in understanding disease progression and for considering potential points of intervention, while still recognizing the limits of the current evidence [8]. The literature focused on gerontology reaches a similar conclusion from a broader perspective: that epigenetic patterns change over time, and these changes often involve genes linked to metabolism and stress response [9,10].

The retinal pigment epithelium (RPE) occupies a central position in this discussion, as multiple forms of stress converge within this cell monolayer. The RPE sustains photoreceptor function through daily outer segment phagocytosis, lipid recycling, vectorial transport, and maintenance of the outer blood–retina barrier while operating under conditions of high oxygen tension and light exposure. Mechanistic syntheses of AMD consistently place RPE dysfunction upstream of many secondary retinal changes, particularly in the geographic atrophy AMD [4]. Epigenetic studies centered on the RPE have reported alterations in DNA methylation, histone-associated regulatory pathways, and non-coding RNA control in AMD-relevant settings, supporting the view that regulatory remodeling accompanies progressive RPE decline [11]. From this perspective, genome-wide profiling of chromatin accessibility has been especially informative, as it provides a direct view of regulatory architecture: ATAC-seq analyses of human donor tissue reveal a broad reduction in chromatin accessibility in AMD, with pronounced effects in the RPE [12]. The RPE possesses intrinsic antioxidant defense systems that coordinate cellular responses to oxidative stress and maintain redox homeostasis.

Although previous reviews have discussed AMD genetics, RPE dysfunction, mitochondrial impairment, oxidative stress, or epigenetic regulation separately, fewer have integrated these processes into a unified redox-sensitive regulatory framework. The novelty of this review lies in positioning redox imbalance not merely as a damaging consequence of aging or mitochondrial dysfunction but as a central mechanistic axis that connects mitochondrial stress, metabolic remodeling, and epigenetic regulation in the RPE. In this context, oxidative stress is considered both a source of molecular injury and a signaling mechanism capable of influencing DNA methylation, chromatin accessibility, histone modification, and RNA-based regulation. This perspective is particularly relevant to Antioxidants because it emphasizes how antioxidant defense systems and redox-sensitive signaling pathways may shape long-term transcriptional remodeling in AMD.

Several unresolved questions continue to frame the interpretation of epigenetic regulation in AMD. It remains unclear why epigenetic remodeling appears most prominently in the RPE rather than in the neuroretina, whether these changes actively contribute to degeneration or primarily reflect chronic metabolic and oxidative stress, and at what stage such alterations lose reversibility. In this review, we focus on the interaction between epigenetic regulation, mitochondrial dysfunction, cellular metabolism, and redox signaling in the RPE during AMD. We interpret epigenetic changes as stress-responsive regulatory layers that may reflect long-term exposure to metabolic and oxidative stress rather than as isolated initiating events. Throughout the review, we distinguish findings derived directly from human AMD-RPE studies from mechanisms inferred from experimental models or broader aging systems, thereby providing a conceptual framework linking aging, environmental stress, mitochondrial dysfunction, antioxidant defense, and RPE vulnerability across DNA methylation, chromatin remodeling, RNA-based regulation, and mitochondria–epigenome crosstalk.

## 2. The Retinal Pigment Epithelium as an Epigenetically Vulnerable Tissue

The RPE forms a polarized monolayer between the neural retina and the choroid to sustain photoreceptor function throughout life. Studies of RPE biology describe this tissue not simply as a passive support layer but as a site where transport, recycling, and the regulation of stress are coordinated in parallel [13,14]. In functional terms, the RPE operates a continuous maintenance program for the outer retina: it mediates nutrient and ion transport, clears metabolic waste, and preserves the integrity of the outer blood–retina barrier [15,16].

This combined metabolic and phagocytic workload is unusually persistent for a terminally differentiated epithelial tissue. Each RPE cell engulfs and degrades photoreceptor outer segments in a daily rhythm, a process that tightly couples lysosomal activity with lipid processing and ATP demand [16,17]. Strauss’s physiological analysis underscores the uninterrupted nature of this task and explains why even modest declines in efficiency can lead to the development of pathology over time [15]. A related constraint is further imposed, as the adult human RPE exhibits only limited turnover, meaning that accumulated stress and damage are not ameliorated through the generation of new cells, as they would be in more rapidly renewing epithelia [14].

Mitochondria lie at the core of this vulnerability. In human-derived RPE cells, mitochondria are abundant and densely distributed, particularly in the metabolically active basal and perinuclear regions, reflecting the high energetic demand required for ion transport, outer segment phagocytosis, lipid recycling, and barrier maintenance [18,19,20]. Quantitative ultrastructural studies and three-dimensional reconstructions have shown that RPE cells contain a high mitochondrial volume density compared with many other epithelial cell types, supporting the view that mitochondrial integrity is central to long-term RPE homeostasis [21,22]. Reactive oxygen species (ROS) in the RPE originate from multiple sources, including mitochondrial electron transport chain leakage (particularly complexes I and III), NADPH oxidases, and photo-oxidative stress associated with lipofuscin accumulation such as A2E [23,24].

Importantly, mitochondrial abnormalities in AMD have been reported in both human donor tissues and experimental models, although the strength of evidence differs across systems. Human AMD samples provide direct evidence of altered mitochondrial structure, reduced respiratory protein expression, and increased oxidative damage in the RPE, whereas in vitro and animal models provide stronger mechanistic support for how mitochondrial stress may influence redox signaling, metabolic flux, and epigenetic regulation [18,19]. Importantly, ROS act not only as damaging agents but also as signaling molecules that regulate redox-sensitive transcriptional and epigenetic processes. The RPE is equipped with antioxidant systems, which collectively buffer oxidative stress. Therefore, mitochondrial dysfunction should be interpreted as a strongly supported AMD-associated feature, while specific causal pathways linking mitochondrial impairment to epigenetic remodeling remain partly model-dependent.

The RPE also possesses an extensive antioxidant defense network that buffers this chronic oxidative burden. Enzymatic antioxidant systems include superoxide dismutases (SOD1 and SOD2), catalase, glutathione peroxidases, peroxiredoxins, and thioredoxin-related pathways [25,26,27]. In parallel, the glutathione redox couple (GSH/GSSG) provides a major non-enzymatic buffering system that helps maintain intracellular redox balance [25,28]. The Nuclear factor erythroid 2-related factor 2 (NRF2)–Kelch-like ECH-associated protein 1 (KEAP1) pathway is particularly important because it coordinates the transcription of genes involved in antioxidant defense, detoxification, glutathione metabolism, and mitochondrial stress adaptation [26,29,30]. Under physiological conditions, these systems allow the RPE to tolerate high oxygen tension, light exposure, and daily phagocytic load. During aging and AMD, however, persistent oxidative stress may overwhelm redox buffering capacity, thereby shifting ROS from adaptive signaling molecules toward sustained mediators of cellular injury and regulatory remodeling [18,19,26].

The post-mitotic nature of the RPE changes how epigenetic alterations should be interpreted. In proliferative tissues, some regulatory changes may be ameliorated through successive rounds of cell division. In contrast, epigenetic markers in the RPE can persist for decades, giving even subtle, locus-specific shifts sufficient time to exert biological effects. Retina-focused epigenetic reviews have noted that epigenetic alterations associated with AMD tend to be selective rather than global, with signals clustering in proximity to genes involved in stress regulation, extracellular matrix organization, and metabolism [7,31].

Lipid handling imposes an additional, parallel burden. The aging Bruch’s membrane microenvironment may further influence the epigenetic state of the RPE. With age, Bruch’s membrane undergoes progressive thickening, lipid accumulation, extracellular matrix remodeling, and increased stiffness, all of which can impair nutrient exchange and metabolic coupling between the choroid and the RPE [32,33,34]. These changes may impose chronic metabolic, oxidative, and mechanical stress on RPE cells, thereby altering mitochondrial function, redox balance, and stress-response signaling [33,35]. In addition, extracellular matrix remodeling may influence cell adhesion, mechanotransduction pathways, and cytoskeletal organization, which are increasingly recognized as factors capable of affecting chromatin organization and transcriptional regulation [34,36]. Although direct evidence linking aging Bruch’s membrane to specific epigenetic modifications in human AMD-RPE remains limited, the altered extracellular environment likely contributes to the broader stress landscape within which epigenetic remodeling occurs [32,35].

The RPE processes large amounts of lipid-rich photoreceptor outer segment material and plays a central role in regulating cholesterol movement within the retina [37]. Detailed pathological studies have shown that aging eyes gradually accumulate lipid-associated deposits, along with structural changes in Bruch’s membrane and the sub-RPE space, features that align with early stages of AMD [38]. Studies of retinal cholesterol metabolism further suggest that disruptions in lipid flux may contribute to oxidative stress and altered cellular signaling in ways that become difficult to reverse once established [39]. Drusen represent a clinically visible manifestation of this slow process, but the underlying cellular etiology originates much earlier and largely escapes direct observation [38]. Environmental exposure places additional pressure on these mechanisms. Smoking is a well-established epidemiological risk factor and introduces oxidative and inflammatory stress that further tests the resilience of RPE cells. Experimental studies centered on the RPE have linked exposure to cigarette smoking with impaired autophagy and altered stress-response pathways, suggesting further mechanistic insights that fit the slow time course of AMD progression [40]. Systems-level analyses of the geographic atrophy AMD place this type of chronic stress within a wider network that also includes metabolic disruption, inflammation, and tissue remodeling [41]. Viewed from this perspective, early chromatin alterations observed in human RPE datasets are therefore not unexpected, as chromatin structure represents one way that cells retain a record of prolonged stress exposure [12].

Together, these features indicate that the RPE is not only metabolically active but also redox-sensitive. Its high mitochondrial density, continuous phagocytic workload, and exposure to light and oxygen generate a persistent requirement for antioxidant defense. When mitochondrial function declines or antioxidant buffering becomes insufficient, redox imbalance may influence epigenetic regulation through changes in NAD^+^ availability, acetyl-CoA metabolism, DNA methylation capacity, and histone-modifying enzyme activity. This provides a mechanistic basis for considering the RPE as an epigenetically vulnerable tissue in AMD. However, while mitochondrial and oxidative abnormalities are well supported in human AMD RPE, detailed mechanistic links between redox imbalance and specific epigenetic modifications remain more strongly supported by experimental models and require further validation in human tissue.

## 3. DNA Methylation and Metabolic Gene Regulation in RPE Cells of AMD Patients

Among the epigenetic mechanisms discussed in AMD, DNA methylation features most consistently in human data, particularly when analyses focus directly on the RPE [42,43]. In this tissue, methylation carries a practical advantage: it is sufficiently stable to reflect effects of long-term alterations, yet it remains linked to cellular metabolism through shared biochemical cofactors [44,45]. This combination helps explain why DNA methylation continues to feature prominently in AMD pathogenesis, even when the reported effect sizes are relatively modest. Importantly, current DNA methylation evidence in AMD should be interpreted with caution. Most available studies are cross-sectional and based on limited donor numbers or heterogeneous ocular tissues, which restrict conclusions about temporal sequence and causality [46,47,48,49,50]. Reported methylation differences are often modest in effect size and may vary across cohorts, platforms, and tissue sources [7,46]. Therefore, DNA methylation changes in AMD are best viewed as disease-associated regulatory signatures unless supported by matched gene-expression data, functional perturbation, or longitudinal validation. Cellular redox status may influence DNA methylation by modulating the activity of DNA methyltransferases (DNMTs) and ten-eleven translocation (TET) enzymes, thereby linking oxidative stress to epigenetic alterations [48,49,50].

Early studies of DNA methylation in AMD patients relied largely on peripheral blood samples or on data obtained from a limited number of candidate loci. These reports identified reproducible differences between patients and controls, supporting the presence of systemic epigenetic signals [42]. What remained unclear was the extent to which these systemic signals act directly within ocular tissues. This uncertainty shifted attention toward studies of the eye itself, where mechanistic inferences can be made in a more disease-relevant context.

Genome-wide profiling of postmortem RPE has since presented a more focused view of epigenetic changes. Differences in DNA methylation between AMD vs. control samples are not distributed evenly across the genome but instead cluster at specific loci, often near genes involved in transcriptional control, DNA repair, extracellular matrix organization, and stress response pathways [43]. Analysis of these patterns does not reveal widespread hypomethylation of repetitive elements, which therefore argues against a model of global epigenomic collapse. Rather, it points towards a selective regulatory remodeling shaped by RPE stress. For this reason, studies that align methylation changes with gene expression are particularly informative. In a post-mitotic tissue, even modest regulatory shifts can carry significant weight, because these persist over time and are not offset by cell replacement. A key limitation is that most methylation studies identify correlations rather than functional causality. Differential methylation near genes involved in stress response, extracellular matrix regulation, or mitochondrial maintenance does not necessarily demonstrate that methylation directly controls these pathways. Instead, such patterns may reflect prior exposure to oxidative stress, inflammation, altered cellular composition, or late-stage tissue remodeling. Functional validation using targeted methylation editing, methylation-sensitive reporter assays, or matched methylome–transcriptome analyses in AMD-relevant RPE models will be required to determine whether these changes actively regulate gene expression or mainly serve as molecular records of disease exposure. This view aligns with findings from models of retinal degeneration, where disease severity tends to follow graded changes in transcription rather than binary on–off transitions [51].

Age provides an additional lens through which to interpret these findings. Across multiple tissues, DNA methylation patterns change with age in ways that can be modeled as epigenetic “clocks”, and these patterns often map to regulatory regions linked to cellular maintenance, metabolic control, and stress responses [31,52]. Studies in the retina similarly describe methylation as one of several regulatory layers that shift with age and progressively reshape cellular programs [31]. The ELOVL2 locus is frequently cited as a robust age-associated methylation marker, and reduced ELOVL2 activity has been linked in experimental settings to altered lipid processing and cellular stress phenotypes [53]. However, its functional significance in AMD remains poorly established. In the RPE, where lipid handling is a sustained physiological burden, ELOVL2 provides a useful example of age-related metabolic epigenetic regulation, but current evidence does not support its interpretation as a validated driver of AMD-RPE degeneration.

More broadly, the relevance of DNA methylation in AMD may extend beyond ELOVL2 itself to include wider lipid-handling and cholesterol-regulatory pathways within the RPE. The RPE occupies a central position in retinal lipid metabolism because it continuously processes photoreceptor outer segments, circulating lipids, and fatty acid intermediates under conditions of high oxidative and metabolic demand [54,55]. In this context, epigenetic regulation of lipid-associated genes such as APOE and ABCA1 may also be relevant to AMD biology, given their established roles in lipid transport, cholesterol homeostasis, drusen composition, and inflammatory signaling [56,57,58]. Although direct evidence for locus-specific methylation-mediated regulation of these genes in human AMD-RPE remains limited, available findings support the broader concept that chronic metabolic and oxidative stress may reshape transcriptional programs involved in lipid handling through epigenetic mechanisms [46,48,59]. Thus, ELOVL2 should be interpreted not as an isolated epigenetic event but as part of a larger metabolically sensitive regulatory network associated with aging and RPE stress adaptation.

The biochemical link between cellular metabolism, redox state, and DNA methylation is direct but context-dependent. DNMTs require S-adenosylmethionine (SAM) as a methyl donor, while SAM availability depends on one-carbon metabolism, folate cycling, methionine metabolism, and mitochondrial function [48,60,61]. Chronic oxidative stress may influence this axis by altering NAD^+^/NADH balance, mitochondrial metabolite production, and the availability of reducing equivalents required for one-carbon flux [25,36,48]. In addition, redox imbalance may affect the activity of methylation-regulatory enzymes, including DNMTs and TET dioxygenases [25,46]. TET enzymes require Fe^2+^, α-ketoglutarate (α-KG), and oxygen, making their activity potentially sensitive to mitochondrial metabolism, oxidative stress, and cellular redox state [46,49]. These mechanisms provide a plausible route through which mitochondrial dysfunction and ROS signaling may influence locus-specific methylation patterns in the RPE.

Cellular redox status can influence one-carbon metabolism and SAM availability, placing DNA methylation under redox-sensitive metabolic control. DNMT activity depends on SAM availability, which is shaped by one-carbon metabolism and mitochondrial function. Studies of DNA methylation alongside those investigating one-carbon metabolism show that methylation capacity may in fact be constrained by metabolic state [45,62]. From a broader perspective, when considered across multiple systems, it appears that metabolism and epigenetic regulation are coupled through shared substrates and cofactors, such that chronic metabolic stress can influence the direction of regulatory change [53,63].

**Figure 1:**
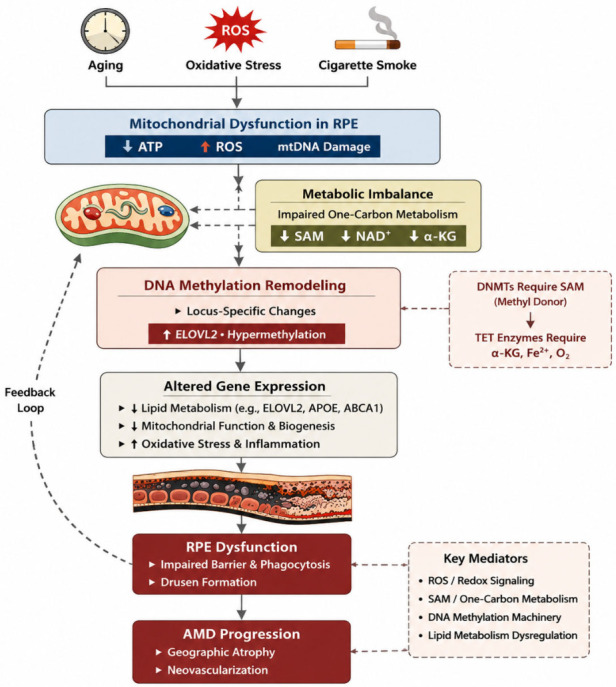
Metabolic control of DNA methylation in the retinal pigment epithelium during AMD. Aging, oxidative stress, cigarette smoke exposure, and other environmental stressors contribute to mitochondrial dysfunction in retinal pigment epithelium (RPE) cells. Mitochondrial impairment is associated with reduced ATP production, increased reactive oxygen species (ROS) generation, mitochondrial DNA (mtDNA) damage, and altered metabolic output. These changes may disrupt one-carbon metabolism and cellular redox homeostasis, resulting in altered availability of key metabolic cofactors involved in DNA methylation regulation, including S-adenosylmethionine (SAM), nicotinamide adenine dinucleotide (NAD^+^) and TET α-ketoglutarate (α-KG). DNA methyltransferases (DNMTs) require SAM as a methyl donor, whereas ten-eleven translocation (TET) enzymes depend on α-KG, Fe^2+^, and oxygen for active DNA demethylation. Altered metabolic and redox states may therefore influence locus-specific DNA methylation patterns. ELOVL2 hypermethylation is shown as an illustrative example of age-associated epigenetic remodeling because it represents one of the most reproducible methylation signatures associated with biological aging. However, its direct functional contribution to AMD pathogenesis remains incompletely established. Changes in DNA methylation may influence transcriptional programs involved in lipid metabolism, mitochondrial maintenance, oxidative stress responses, and inflammatory signaling, thereby contributing to progressive RPE dysfunction. Impaired barrier integrity, defective phagocytosis, drusen formation, and chronic inflammatory activation may subsequently promote AMD progression. A potential feedback relationship is proposed whereby persistent mitochondrial dysfunction and oxidative stress may further reinforce epigenetic remodeling over time. Solid arrows indicate interactions supported by experimental evidence from human AMD tissues and/or experimental models. Dashed arrows indicate proposed, indirect, or incompletely validated mechanisms. The figure summarizes current evidence and conceptual models discussed in this review and should not be interpreted as a definitive causal sequence. Schematic illustrations were created and assembled by the authors using Adobe Illustrator.

Environmental exposure can also affect DNA methylation in the same direction. For example, smoking represents a strong epidemiologic risk factor for AMD and is associated with reproducible methylation signatures in human studies [64]. In AMD patients, partial overlap between blood-based markers and methylation changes in the retina has been reported, supporting the idea that systemic exposure can leave detectable epigenetic traces while still allowing for tissue-specific regulatory outcomes [42]. In experimental RPE cell models, smoke-induced oxidative stress engages pathways that intersect with methylation-sensitive gene regulation, providing a cellular route that links epidemiologic observations to molecular change [40,65]. These findings support a model in which DNA methylation functions as a context-dependent regulatory mechanism (Figure 1). Rather than acting as an independent trigger of AMD, methylation integrates aging, metabolic state, mitochondrial function, and environmental exposure into sustained transcriptional shifts that shape disease trajectory over time. Most of these findings are based on association studies in human donor tissues or experimental models rather than direct functional validation.

Taken together, DNA methylation in AMD should be interpreted as a selective and metabolically sensitive regulatory layer rather than as evidence of global epigenomic failure. Human RPE methylation studies provide important disease-associated signals, but their modest effect sizes, cross-sectional design, and limited functional validation constrain causal interpretation. The strongest current model is that aging, mitochondrial dysfunction, oxidative stress, and altered one-carbon metabolism may converge to shape locus-specific methylation states, which may then contribute to sustained transcriptional bias in the RPE. Future studies should integrate methylome, transcriptome, chromatin accessibility, and metabolomic data from matched human RPE samples and should include functional validation in AMD-relevant RPE systems. However, there is on-going debate over whether changes in DNA methylation serve as primary drivers of RPE degeneration or instead reflect downstream responses to chronic metabolic and oxidative stress. Several studies note that most methylation differences reported in AMD patients are modest in scale and are derived from cross-sectional donor tissues, a design that inherently limits causal inference [42,43]. DNA methylation may operate less as an initiating event and more as a regulatory layer that helps stabilize transcriptional states once cellular stress is already in place. Resolving these differences will require scrutiny of longitudinal human data, which are still largely absent from current AMD studies.

## 4. Chromatin Accessibility and Histone Modifications in RPE Dysfunction

DNA methylation often serves as a long-term record of exposure, whereas chromatin structure reflects how such exposure is translated into day-to-day regulation of gene expression. Changes in chromatin accessibility and histone modifications can occur on shorter time scales and respond to stress without requiring cell division. Conceptual frameworks of histone regulation emphasize this point, describing chromatin not merely as a structural scaffold but as an active layer of gene regulation [66]. In aging tissues, shifts in histone acetylation have also been linked with altered stress responses and reduced transcriptional flexibility [67]. These principles are highly relevant in the RPE, where cells must preserve core gene programs regulating homeostatic activity over decades while remaining responsive to repeated and transient oxidative and metabolic challenges [13,18].

Direct evidence for chromatin remodeling associated with AMD comes from genome-wide analyses of chromatin accessibility. ATAC-seq profiling of human donor eyes revealed reduced chromatin accessibility in AMD tissues, with some of the most pronounced changes observed in the RPE relative to the neural retina [12]. This human donor ATAC-seq evidence provides important support for altered regulatory architecture in AMD-RPE. However, interpretation of these findings requires caution. Bulk tissue ATAC-seq analyses may be influenced by tissue heterogeneity, including mixtures of relatively preserved and degenerating RPE cells, infiltrating immune cells, and other retinal cell populations present within AMD-affected tissue [68]. Consequently, reduced accessibility signals may reflect not only intrinsic chromatin remodeling within stressed RPE cells but also shifts in cellular composition and disease-stage variability.

In addition, a broad decrease in chromatin accessibility does not necessarily imply a uniform loss of transcriptional capacity across the genome. Rather, it may indicate selective remodeling of regulatory regions associated with oxidative stress responses, mitochondrial maintenance, lipid metabolism, inflammatory signaling, and RPE identity. These findings therefore support the interpretation that chromatin remodeling in AMD follows functional pathways already central to RPE biology rather than representing nonspecific global regulatory collapse. Importantly, while human donor studies identify disease-associated chromatin states, mechanistic explanations for how oxidative stress, mitochondrial dysfunction, altered metabolite availability, or chronic inflammatory stress reshape chromatin accessibility remain largely inferred from experimental RPE models and broader chromatin biology.

Viewed in this context, chromatin accessibility changes in AMD are more plausibly interpreted as stress-responsive regulatory adaptations that may progressively become less reversible with aging and chronic metabolic stress. However, because bulk epigenomic approaches cannot fully resolve cell-type-specific contributions, future studies using single-cell or spatial epigenomic technologies will be essential to distinguish intrinsic RPE chromatin remodeling from secondary effects related to retinal degeneration, immune-cell infiltration, or tissue heterogeneity.

Interestingly, histone acetylation offers a mechanistic link between cellular stress and chromatin accessibility. Acetylation is generally associated with a more open chromatin state that supports transcription, whereas deacetylation favors chromatin compaction and limits regulatory access. Reviews of chromatin regulation describe this balance as a central means by which cells adjust gene expression in response to changing conditions [42]. In the RPE, such tuning is likely to be tightly coupled to metabolic state, since histone acetylation depends on the availability of acetyl-CoA, a metabolite whose supply is shaped by mitochondrial function [63,69,70]. Redox-dependent metabolic changes may influence histone modifications through cofactors such as acetyl-CoA and NAD^+^, thereby linking mitochondrial function and chromatin regulation.

Beyond overall acetylation status, specific histone marks may provide more precise insight into AMD-associated chromatin states. Histone H3 lysine 27 acetylation (H3K27ac) is commonly associated with active enhancers and transcriptionally permissive regulatory elements, whereas H3K9me3 is linked to repressive heterochromatin and long-term transcriptional silencing [71,72]. In the RPE, altered balance between permissive marks such as H3K27ac and repressive marks such as Histone H3 lysine 9 trimethylation (H3K9me3) may influence the accessibility of genes involved in antioxidant defense, mitochondrial quality control, lipid metabolism, and inflammatory signaling [8,72]. However, direct mapping of these marks in human AMD-RPE remains limited, and future studies integrating ATAC-seq with ChIP-seq or CUT&Tag will be needed to define their locus-specific roles [68,73].

Studies in AMD-related contexts have reported altered expression of histone-modifying enzymes, with particular attention to histone deacetylases (HDACs). For instance, work in RPE models and in AMD tissue has described increased HDAC expression under conditions of oxidative stress [74,75]. Elevated HDAC activity is consistent with reduced chromatin accessibility, as enhanced deacetylation is expected to promote chromatin compaction and reduce access to enhancers/promoters that support stress-response as well as metabolic gene programs. In a post-mitotic tissue such as the RPE, these regulatory shifts can persist for long periods, which may be sufficient to influence how cells respond to subsequent insults. Sirtuins, particularly NAD^+^-dependent deacetylases such as Sirtuin 1 (SIRT1) and Sirtuin 3 (SIRT3), serve as key mediators linking mitochondrial metabolism, redox state, and histone deacetylation in the RPE [76]. These findings are largely derived from experimental models and require validation in human AMD-RPE.

Increased HDAC activity could plausibly contribute to reduced histone acetylation and chromatin compaction, but this interpretation remains partly model-dependent and incompletely validated in human AMD-RPE [8,77]. Intervention studies provide functional support for this view, while also making their limitations clear. In experimental models of retinal degeneration, inhibition of histone deacetylases has been shown to confer protection, improving stress tolerance and partially restoring gene expression patterns consistent with chromatin relaxation [78]. At the same time, important caveats remain for therapeutic approaches based on HDAC inhibition. Because HDACs are broadly expressed, non-selective HDAC inhibitors raise concerns about target specificity, dose-dependent effects, and long-term safety. Even so, these findings indicate that chromatin organization in stressed retinal cells retains a degree of plasticity rather than being irreversibly fixed.

Nevertheless, HDAC inhibition should not be interpreted as a straightforward therapeutic solution for AMD. HDACs regulate broad transcriptional programs across multiple retinal and non-retinal cell types, and non-selective inhibition may produce off-target effects, altered inflammatory responses, or unwanted changes in cell identity [8,79]. Safety is also a concern because chronic modulation of histone acetylation may affect genes unrelated to AMD pathology. More selective HDAC isoform targeting, local delivery, and stage-specific treatment design will be necessary before HDAC-based approaches can be considered clinically feasible [8,79].

Metabolic state repeatedly emerges as a background condition that shapes chromatin regulation. The availability of acetyl-CoA links mitochondrial output to histone acetylation, and broader reviews on metabolic control of chromatin suggest that this connection can bias transcriptional programs under conditions of chronic stress [63,70]. For example, in aging RPE, where mitochondrial defects are well documented, reduced acetyl-CoA supply offers a plausible route toward a more deacetylated and less accessible chromatin state [18,19]. Once established, such transcriptional restriction may further compromise mitochondrial maintenance, creating a feedback loop that is consistent with findings from AMD-related transcriptomic studies, particularly those showing coordinated disruption of metabolic and mitochondrial gene programs [18,80]. Redox-sensitive chromatin regulators provide another mechanistic link between oxidative stress and epigenetic remodeling. Sirtuins, particularly NAD^+^-dependent deacetylases such as SIRT1 and SIRT3, connect cellular redox state to histone deacetylation, mitochondrial function, and stress adaptation [76]. Poly(ADP-ribose) polymerase (PARP) enzymes may also influence chromatin regulation through NAD^+^ consumption during DNA damage responses, thereby linking oxidative injury to changes in chromatin-associated metabolism [81]. In addition, oxidative stress may indirectly affect histone acetyltransferase and methyltransferase activity by altering cofactor availability, mitochondrial metabolite output, and nuclear redox balance [25,49]. These mechanisms support the idea that chromatin remodeling in the RPE may be shaped by redox-sensitive metabolic signaling rather than by chromatin enzymes alone.

Histone markers beyond acetylation may also contribute to stress-associated chromatin remodeling in the RPE, although direct evidence from human AMD donor tissues remains limited. Studies in models of retinal degeneration link disease progression with broader shifts in histone modification patterns, suggesting that multiple histone markers can change as retinal cells respond to persistent oxidative and metabolic stress [82]. An important next step in AMD studies will be to clearly map these modifications in the RPE, allowing chromatin accessibility, histone state, and transcriptional output to be examined together within the same tissue framework [12].

Collectively, current evidence supports a model in which chromatin accessibility and histone modifications act as stress-responsive regulatory layers in the RPE, but the strength of evidence differs across mechanisms. Human AMD donor studies provide direct evidence for altered chromatin accessibility, whereas mechanistic links involving HDAC activity, specific histone marks, mitochondrial metabolites, and redox-sensitive chromatin regulators remain more dependent on experimental models and require further validation. At earlier disease stages, chromatin remodeling may represent an adaptive response to oxidative and metabolic stress. With persistent aging-related stress, however, these changes may become less reversible and contribute to sustained transcriptional bias. Importantly, chromatin-based regulation should be viewed as part of an integrated DNA methylation–metabolism–redox network rather than as an isolated causal pathway.

## 5. Mitochondria–Epigenome Crosstalk in the RPE

**Figure 2:**
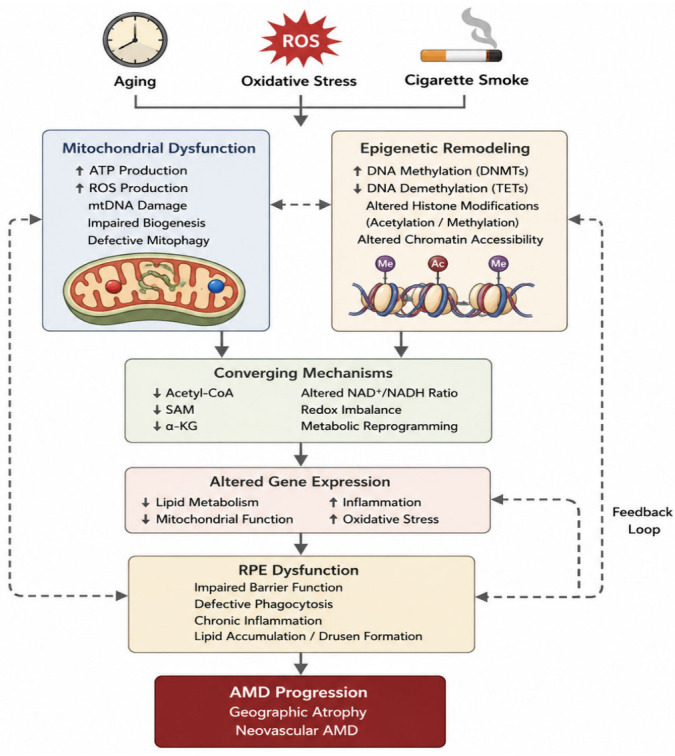
Mitochondrial dysfunction–epigenetic crosstalk in the RPE during AMD progression. Aging, oxidative stress, cigarette smoke exposure, and other chronic environmental stressors contribute to mitochondrial dysfunction and epigenetic remodeling in retinal pigment epithelium (RPE) cells. Mitochondrial abnormalities observed in AMD are characterized by impaired oxidative phosphorylation, defective mitophagy, altered mitochondrial biogenesis, mitochondrial DNA (mtDNA) damage, and increased reactive oxygen species (ROS) production. In parallel, epigenetic alterations, including changes in DNA methylation, histone modifications, and chromatin accessibility, may influence the transcriptional programs that regulate cellular stress responses, mitochondrial maintenance, and metabolic homeostasis. Mitochondrial dysfunction and epigenetic remodeling are proposed to interact through a bidirectional regulatory network. Mitochondrial impairment may alter the availability of key metabolic cofactors, including acetyl-CoA, nicotinamide adenine dinucleotide (NAD^+^), S-adenosylmethionine (SAM), and α-ketoglutarate (α-KG), thereby influencing DNA methylation, histone acetylation, chromatin organization, and transcriptional regulation. Conversely, epigenetic alterations may affect the expression of genes involved in mitochondrial biogenesis, oxidative phosphorylation, antioxidant defense, lipid metabolism, and mitophagy, potentially influencing mitochondrial function and stress adaptation. The convergence of mitochondrial dysfunction and epigenetic remodeling may promote metabolic reprogramming, redox imbalance, chronic inflammatory signaling, and altered gene-expression profiles. These changes are associated with impaired barrier function, defective phagocytosis, lipid accumulation, drusen formation, and progressive RPE dysfunction, ultimately contributing to AMD progression, including geographic atrophy and neovascular AMD. A self-reinforcing feedback relationship is proposed whereby persistent oxidative and metabolic stress may progressively strengthen mitochondria–epigenome interactions over time. Human AMD donor studies provide direct evidence for mitochondrial abnormalities and altered chromatin accessibility, whereas many mechanistic links involving metabolite-dependent epigenetic regulation, mitonuclear signaling, and feedback interactions are derived primarily from experimental RPE models, cybrid systems, animal studies, or broader aging-related literature. Solid arrows indicate interactions supported by experimental evidence. Dashed arrows indicate proposed, indirect, or incompletely validated mechanisms. The figure summarizes current evidence and conceptual models discussed in this review and should not be interpreted as a definitive causal sequence. Schematic illustrations were created and assembled by the authors using Adobe Illustrator.

The following section integrates findings from both human studies and experimental systems; distinctions are explicitly noted where applicable. Mitochondrial dysfunction accompanied by persistent redox imbalance is one of the most consistent features reported in AMD and is often most evident within the RPE. This statement is supported by multiple lines of AMD-specific evidence, including analyses of human donor RPE, AMD-relevant RPE cultures, and retinal degeneration models. Human AMD samples have shown altered mitochondrial morphology, impaired respiratory protein expression, reduced oxidative phosphorylation capacity, and increased oxidative damage in the RPE [18,19,20]. These observations provide direct disease relevance, although most human studies capture late or endpoint pathology rather than the temporal sequence of disease progression. Experimental RPE models, by contrast, provide stronger mechanistic insight into how mitochondrial impairment may influence ROS production, metabolite availability, and nuclear gene regulation, but they do not fully reproduce the chronic and spatially heterogeneous environment of the aging human macula. The proposed bidirectional interactions between mitochondrial dysfunction, metabolic remodeling, and epigenetic regulation in AMD-RPE are summarized in Figure 2.

Studies centered on RPE mitochondria report reduced respiratory capacity, altered mitochondrial architecture, and increased oxidative damage, with changes that can be detected prior to late-stage retinal degeneration [18,65]. Mitochondria operate well beyond their role as producers of ATP. The mitochondrial redox state functions not only as an indicator of cellular stress but also as an active regulator of nuclear gene expression programs, reflecting the broader role of mitochondria as signaling organelles that convey cellular status to the nucleus [83]. In the setting of aging and chronic diseases such as AMD, this signaling dimension becomes particularly relevant, as the same metabolic pressures that impair mitochondrial output also reshape the availability of cofactors required for epigenetic regulation [63,84].

One way to view this relationship is to consider metabolites rather than genes. Mitochondria help determine the availability of acetyl-CoA and NAD^+^, metabolites that directly modulate chromatin regulators such as histone acetyltransferases and sirtuins. Studies linking metabolism to epigenetic control emphasize that these interactions are not secondary effects but rather part of the core machinery by which cells translate energy status into transcriptional output [63,80]. In the RPE, mitochondrial function is integral to the metabolic demands of continuous transport activity and the daily phagocytosis of shed photoreceptor outer segments. This high dependence on oxidative phosphorylation means that even modest declines in mitochondrial efficiency may alter acetyl-CoA availability. Such shifts are relevant to epigenetic regulation because they can influence histone acetylation and, in turn, reshape chromatin accessibility at metabolic gene loci [19,69,70]. Seen in this light, mitochondrial dysfunction may contribute not only to metabolic insufficiency but also to the chromatin-level remodeling that progressively undermines RPE homeostasis in AMD.

In addition to histone acetylation, histone methylation may also participate in mitochondria–epigenome crosstalk in the RPE. Methylation marks on histones H3 and H4, including activating marks such as H3K4me3 and repressive marks such as H3K9me3 or H3K27me3, can influence chromatin accessibility and transcriptional stability at metabolic and stress-response loci [71,72]. Mitochondrial dysfunction may affect these marks indirectly by altering metabolite availability. For example, SAM provides methyl groups for histone methyltransferases, whereas α-ketoglutarate-dependent demethylases are sensitive to mitochondrial metabolic state and may be inhibited by accumulated succinate or fumarate [48,49]. In this way, impaired mitochondrial metabolism may influence not only acetylation/deacetylation balance through acetyl-CoA and NAD^+^ but also histone methylation dynamics through methyl-donor and tricarboxylic acid cycle intermediates. Although such mechanisms are highly plausible in the RPE, direct evidence linking specific H3/H4 methylation changes to mitochondrial dysfunction in human AMD-RPE remains limited.

Redox signaling provides a second route linking mitochondrial state to gene regulation. Mitochondria represent a major source of ROS, which may induce cellular damage but also function as signaling mediators regulating redox-sensitive transcriptional and epigenetic pathways. Studies of mitochondrial ROS indicate that changes in ROS levels can modulate the activity of transcription factors and reshape stress-response programs [85]. In the RPE, prolonged oxidative pressures can therefore promote genetic programming toward a sustained “defense mode”, a state that may support short-term survival while gradually constraining metabolic flexibility over time [18,38]. Mitochondrial ROS may modulate epigenetic regulators, including histone acetyltransferases and deacetylases, thereby potentially influencing chromatin accessibility and gene expression [48]. Under these conditions, epigenetic regulation may help retain a molecular record of prior stress exposure, even after the initiating stress conditions have subsided.

ROS-mediated signaling provides a particularly important route through which mitochondria may influence chromatin regulation. Moderate or compartmentalized ROS signals can activate redox-sensitive transcription factors, including NRF2, NF-κB, AP-1, and HIF-1α, which may subsequently reshape chromatin accessibility at antioxidant, inflammatory, metabolic, and stress-response loci [26,27]. ROS can also affect chromatin indirectly by altering NAD^+^ metabolism, inducing DNA damage responses, and activating PARP-dependent chromatin-associated repair pathways [76,81]. In addition, oxidative stress may influence the activity of DNMTs, TET enzymes, histone acetyltransferases, HDACs, and sirtuins through changes in cofactor availability and cellular redox state [25,49]. These mechanisms provide a direct conceptual bridge between mitochondrial dysfunction, redox signaling, and epigenetic remodeling in the RPE.

Findings from trans-mitochondrial cybrid models further support the importance of mitochondria-to-nucleus signaling. In these systems, cells share an identical nuclear genome but harbor mitochondria derived from different donors, allowing mitochondrial state to be examined in relative isolation. Studies using RPE cybrids have shown that mitochondria from AMD donors can alter nuclear gene expression programs associated with inflammation, oxidative stress responses, and epigenetic regulation [86,87]. This experimental design has clear limitations and does not capture the full complexity of the tissue environment. Even so, this approach addresses a specific question: whether mitochondrial properties may be sufficient to influence nuclear regulatory behavior. The observed effects support this intriguing possibility and are consistent with the broader concepts of mitonuclear communication [84].

Cybrid models are valuable because they allow mitochondrial background to be studied while controlling for nuclear genetic variation. This makes them particularly useful for testing whether AMD-associated mitochondrial haplotypes or donor-derived mitochondria can alter nuclear gene-expression patterns [53,88]. However, these systems have important limitations. They do not fully capture the polarity, phagocytic load, extracellular matrix interactions, immune environment, or spatial metabolic gradients of native RPE tissue. In addition, cybrid models cannot reproduce the long-term aging process or chronic complement-rich microenvironment characteristic of AMD. Therefore, cybrid findings should be interpreted as evidence for mitonuclear signaling potential rather than as definitive proof of causal mitochondrial regulation in human AMD.

However, an as-yet-unresolved question concerns the directionality of signaling in this paradigm. Mitonuclear signaling models often place mitochondrial dysfunction upstream of epigenetic remodeling [65], whereas alternative frameworks emphasize age-associated epigenetic drift as a potential precursor that increases susceptibility to later mitochondrial failure. At present, data from AMD-relevant RPE systems are insufficient to verify the temporal order of these processes with any certainty. Studies of human tissues capture disease endpoints rather than progression, leaving open the question of whether mitochondrial redox imbalance initiates epigenetic change or whether gradual regulatory drift instead destabilizes redox homeostasis over time.

Mitonuclear signaling also offers a way to understand how regulatory changes can become self-reinforcing in a post-mitotic tissue. Studies of mitonuclear communication describe feedback loops in which mitochondrial stress reshapes nuclear transcription, and the resulting transcriptional changes then alter the capacity to homeostatically maintain mitochondria [66,89]. The RPE is particularly susceptible to this dynamic, given its high mitochondrial load coupled with its limited capacity for cell replacement and the need to sustain function under continuous metabolic demand [21,22]. Under such conditions, epigenetic repression of genes involved in mitochondrial maintenance may further impair mitochondrial output, which in turn alters cofactor availability and redox signaling, thereby potentially reinforcing a mitochondrial–epigenetic feedback loop between mitochondrial dysfunction and epigenetic repression over time [18,63].

Metabolism-centered analyses of AMD identify mitochondrial function as a tractable node that connects oxidative stress, inflammation, and cellular aging [90,91]. In principle, stabilizing mitochondrial output and metabolite availability could allow epigenetic regulation to shift toward a more flexible state, without the need to directly target chromatin-modifying enzymes. This possibility remains hypothetical in the context of the RPE but aligns with the broader perspectives in medicine that place mitochondria upstream of regulatory programs governing multiple chronic diseases [80]. From a translational perspective, this bidirectional model has important implications. If mitochondrial dysfunction occurs upstream, interventions aimed at improving mitochondrial quality control, NAD^+^ metabolism, or redox buffering may preserve epigenetic flexibility before irreversible transcriptional remodeling occurs [76,92]. If epigenetic dysregulation precedes mitochondrial failure, then therapies targeting chromatin state or stress-responsive transcriptional programs may be required. In either case, therapeutic timing is likely to be critical, because late-stage AMD tissues may already contain self-reinforcing mitochondrial–epigenetic feedback loops that are difficult to reverse. Longitudinal human studies and stage-specific RPE models will therefore be essential for identifying tractable intervention windows [8,92].

Collectively, mitochondria–epigenome crosstalk provides a useful framework for understanding how metabolic stress may become embedded into persistent regulatory states in the RPE. However, the current evidence supports an integrative and bidirectional model rather than a definitive causal sequence. Human AMD tissues provide strong evidence for mitochondrial abnormalities and altered regulatory landscapes, while experimental models provide mechanistic support for links involving ROS signaling, metabolite availability, and chromatin regulation. Future studies should integrate mitochondrial functional assays, redox profiling, chromatin accessibility, DNA methylation, and transcriptomic analysis in matched human or human-relevant RPE systems. Such approaches will be needed to determine whether mitochondrial dysfunction initiates epigenetic remodeling, results from it, or participates in a self-reinforcing pathogenic loop during AMD progression.

## 6. RNA-Based Regulation Within the Epigenetic–Mitochondrial–Redox Axis

RNA-based regulation provides a dynamic and reversible regulatory layer through which RPE cells may adjust gene expression in response to mitochondrial stress, redox imbalance, inflammatory signaling, and metabolic fluctuation. Unlike DNA methylation and chromatin remodeling, which may contribute to longer-term transcriptional memory, RNA-mediated mechanisms can rapidly adjust gene expression in response to mitochondrial stress, oxidative imbalance, inflammatory signaling, and metabolic fluctuation. These mechanisms include microRNAs (miRNAs), long non-coding RNAs (lncRNAs), and RNA modifications such as N6-methyladenosine (m6A). Their relevance to AMD lies not simply in their altered expression but in their ability to modulate mitochondrial quality control, antioxidant defense, inflammatory tone, and the reversibility of stress-induced gene-expression states.

**Figure 3:**
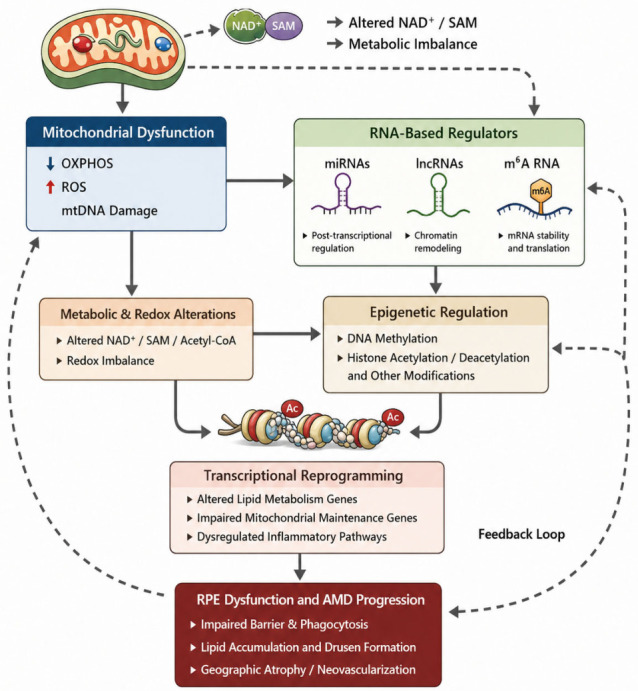
RNA-based regulatory mechanisms linking mitochondrial dysfunction and epigenetic remodeling in retinal pigment epithelium during AMD. Mitochondrial dysfunction in retinal pigment epithelium (RPE) cells is associated with impaired oxidative phosphorylation (OXPHOS), increased reactive oxygen species (ROS) production, mitochondrial DNA (mtDNA) damage, and alterations in cellular metabolism. These changes may disrupt key metabolic cofactors, including nicotinamide adenine dinucleotide (NAD^+^), S-adenosylmethionine (SAM), and acetyl-CoA, thereby influencing RNA-based regulatory pathways and epigenetic processes. RNA-based regulators represent an additional layer of stress-responsive control that may connect mitochondrial dysfunction, metabolic imbalance, redox signaling, and transcriptional adaptation in AMD. MicroRNAs (miRNAs), long non-coding RNAs (lncRNAs), and N6-methyladenosine (m6A) RNA modifications are shown as three major RNA-based regulatory mechanisms. miRNAs primarily regulate post-transcriptional gene expression and may influence mitochondrial dynamics, mitophagy, antioxidant responses, inflammatory signaling, and cellular stress adaptation. lncRNAs may contribute to chromatin-associated regulation and transcriptional control, potentially linking transient stress responses with more persistent regulatory states. m6A RNA modification, mediated by writer, reader, and eraser proteins, may influence mRNA stability, localization, translation efficiency, and stress-responsive protein synthesis under conditions of metabolic and oxidative stress. Metabolic and redox alterations may interact with RNA-based regulators and epigenetic mechanisms, including DNA methylation and histone modifications, thereby contributing to transcriptional reprogramming. Altered expression of genes involved in lipid metabolism, mitochondrial maintenance, oxidative stress responses, and inflammatory signaling may subsequently promote RPE dysfunction. Progressive impairment of barrier integrity, phagocytic function, and cellular homeostasis may contribute to drusen formation, geographic atrophy, and neovascular AMD. A potential feedback relationship is proposed whereby chronic oxidative stress and RPE dysfunction may further reinforce mitochondrial impairment, RNA-based regulatory changes, and epigenetic remodeling over time. Current evidence supporting RNA-based regulation in AMD is heterogeneous. Much of the available evidence for miRNAs and lncRNAs derives from mixed retinal tissues, serum samples, aqueous humor, or experimental systems rather than purified human AMD-RPE. Similarly, direct evidence linking m6A regulators to AMD-specific RPE pathology remains limited. Therefore, RNA-based mechanisms should currently be interpreted as biologically plausible modulators of mitochondrial adaptation and stress responses rather than established drivers of AMD progression. Solid arrows indicate interactions supported by experimental evidence from human tissues and/or experimental models. Dashed arrows indicate proposed, indirect, or incompletely validated mechanisms. The figure summarizes current evidence and conceptual models discussed in this review and should not be interpreted as a definitive causal sequence. Schematic illustrations were created and assembled by the authors using Adobe Illustrator.

Among RNA-based mechanisms, miRNAs currently have the strongest experimental support in AMD-related RPE stress models, particularly in pathways involving oxidative stress, autophagy, inflammation, and mitochondrial homeostasis. Altered miRNA profiles have been reported in serum, aqueous humor, mixed retinal tissue, and experimental RPE models, with associations involving pathways related to apoptosis, inflammation, oxidative stress, mitochondrial maintenance, autophagy, and antioxidant responses; however, much of the current evidence is derived from heterogeneous tissue sources or circulating biomarkers rather than from purified human AMD-RPE [93,94,95,96]. However, much of the current evidence is derived from heterogeneous tissue sources or circulating biomarkers rather than from purified human AMD-RPE. Therefore, these associations should be interpreted cautiously, as altered miRNA expression may reflect broader retinal stress responses, inflammatory signaling, or changes in cellular composition rather than direct RPE-specific regulatory mechanisms. The proposed interactions between mitochondrial dysfunction, RNA-based regulation, epigenetic remodeling, and transcriptional adaptation in AMD-RPE are summarized in Figure 3.

Functionally, miRNAs are more plausibly viewed as network-level modulators that fine-tune RPE stress adaptation by regulating mitochondrial quality control, mitophagy, NRF2-related antioxidant signaling, and inflammatory pathways, rather than as independent AMD initiators [97]. miRNAs linked to mitochondrial dynamics, mitophagy, NRF2-related antioxidant pathways, or inflammatory signaling may influence how RPE cells respond to chronic oxidative and metabolic stress [98,99]. Nevertheless, direct evidence demonstrating causal miRNA-mediated regulation of AMD progression in human RPE remains limited and requires further validation using cell-type-specific and longitudinal approaches.

Among RNA-based mechanisms, miRNA regulation currently has the strongest experimental support in AMD-relevant RPE systems. In particular, oxidative stress-induced miRNA changes have been linked to RPE survival, autophagy, mitochondrial homeostasis, and inflammatory signaling [93,94,95,96]. By contrast, lncRNA- and m6A-related mechanisms remain more exploratory in AMD and require stronger validation using human AMD-RPE datasets, loss- and gain-of-function experiments, and integration with transcriptomic and epigenomic readouts.

lncRNAs may provide a further interface between acute stress signaling and more persistent chromatin-associated states, but their AMD-RPE-specific functional relevance remains less well established than that of miRNAs. Altered lncRNA expression has been reported in AMD-related ocular tissues and experimental systems [100,101], where associations have been described with inflammatory pathways, oxidative stress responses, and metabolic regulation, but many findings derive from heterogeneous tissues or non-RPE-specific datasets, limiting conclusions about direct RPE regulatory roles. Similarly to miRNA studies, much of the available evidence derives from mixed retinal tissues, serum-based analyses, or experimental models rather than from isolated human AMD-RPE. Consequently, many reported lncRNA alterations remain associative and difficult to assign specifically to the RPE.

At present, the strongest conceptual relevance of lncRNAs to this framework is their potential ability to connect transient stress responses with more persistent transcriptional or chromatin-associated states [102]. However, direct functional evidence linking specific lncRNAs to mitochondrial dysfunction, redox signaling, or epigenetic memory in human AMD-RPE remains limited. Future studies using single-cell, spatial transcriptomic, and RPE-specific functional approaches will be necessary to determine which lncRNA changes represent disease-relevant regulatory mechanisms rather than secondary consequences of retinal degeneration [68,103].

m6A modification adds a post-transcriptional mechanism through which metabolic and redox stress may rapidly influence mRNA stability, translation efficiency, and stress-responsive protein output. m6A regulates mRNA stability, translation, and decay through writer, eraser, and reader proteins [104]. The m6A regulatory system is mediated through coordinated actions of “writer”, “reader”, and “eraser” proteins. Writer complexes, including methyltransferase-like proteins such as METTL3, together with cofactors including WTAP, catalyze m6A deposition on target transcripts. Reader proteins, including YTH-domain-containing proteins such as YTHDF1 and YTHDC1, recognize methylated transcripts and influence mRNA stability, localization, translation efficiency, or decay. In contrast, eraser enzymes such as ALKBH5 and FTO can remove m6A marks, thereby introducing reversibility into RNA methylation dynamics [104,105]. Through these mechanisms, m6A modification may rapidly regulate stress-responsive protein synthesis under conditions of mitochondrial dysfunction, oxidative stress, or altered metabolic state.

Although direct evidence linking m6A regulators to AMD-specific RPE pathology remains limited, findings from other stress-related systems suggest that m6A machinery may modulate mitochondrial adaptation, inflammatory signaling, and oxidative stress responses [104,106]. Therefore, m6A-related mechanisms in the RPE should currently be interpreted as biologically plausible but still insufficiently validated components of the broader epigenetic–mitochondrial–redox framework.

Together, RNA-based regulatory mechanisms should be positioned as short-timescale modulators of RPE stress adaptation within the broader epigenetic–mitochondrial–redox axis, with miRNAs currently supported by stronger AMD-relevant evidence than lncRNA or m6A mechanisms. They do not replace DNA methylation or chromatin remodeling as longer-term regulatory layers but may determine how quickly and flexibly RPE cells respond to mitochondrial stress and redox imbalance. Importantly, current evidence does not support a simple causal model in which RNA regulators independently initiate AMD. Rather, miRNAs, lncRNAs, and m6A modifications are more plausibly involved in shaping the intensity, duration, and reversibility of RPE stress responses.

A more integrated interpretation is therefore that RNA-based regulation acts as a bridge between acute stress signaling and persistent epigenetic remodeling. In early or moderate stress states, RNA-mediated mechanisms may support adaptive responses by adjusting mitochondrial quality control, antioxidant defense, and inflammatory signaling. Under chronic aging-related stress, however, sustained dysregulation of these RNA networks may amplify mitochondrial dysfunction and reinforce maladaptive transcriptional states. Future studies should prioritize purified human AMD-RPE datasets, single-cell or spatial transcriptomic analyses, and gain- or loss-of-function experiments to distinguish disease-relevant RNA regulators from secondary stress markers. To clarify the evidence base supporting this epigenetic–mitochondrial–redox framework, key representative studies are summarized in Table 1, with attention to model system, mechanism, principal findings, limitations, and relevance to AMD.

Overall, Table 1 highlights that direct human AMD-RPE evidence is strongest for DNA methylation, chromatin accessibility, and mitochondrial dysfunction, whereas RNA-based regulation and some therapeutic mechanisms remain more dependent on experimental or inferred evidence.

## 7. Therapeutic Implications, Translational Challenges, and Conclusions

Current therapeutic strategies for AMD mainly address downstream pathological manifestations rather than the upstream regulatory disturbances that gradually compromise RPE homeostasis. In neovascular AMD, anti-VEGF therapy can reduce macular exudation and preserve central vision in many patients, but it does not directly target mitochondrial dysfunction, chronic redox imbalance, or epigenetic remodeling in the RPE. In geographic atrophy, where RPE degeneration and photoreceptor loss progress over years, single-target approaches are unlikely to fully modify disease progression because metabolic dysfunction, inflammation, oxidative stress, complement activation, and tissue remodeling occur concurrently [41,107,108]. These considerations support the need for therapeutic strategies that address earlier disease-regulatory mechanisms rather than late structural consequences alone.

Mitochondrial-targeted therapies represent one potential strategy for intervening upstream. Approaches aimed at improving mitochondrial resilience include mitochondrial antioxidants, NAD^+^ supplementation, activation of sirtuin-dependent pathways, stabilization of mitochondrial dynamics, and enhancement of mitophagy to remove damaged mitochondria [19,76,92]. By restoring mitochondrial output and reducing excessive ROS production, these interventions may indirectly preserve chromatin-regulatory capacity through improved acetyl-CoA availability, NAD^+^ balance, and redox homeostasis. However, mitochondrial dysfunction in AMD is heterogeneous and may differ according to disease stage, genetic background, local inflammatory state, and regional retinal vulnerability. Therefore, mitochondrial-targeted therapy should be explored with caution, using RPE-specific functional readouts, optimized dosing, and biomarkers that reflect mitochondrial recovery rather than relying only on anatomical endpoints.

Antioxidant-based interventions are also highly relevant to this framework, particularly given the central role of redox signaling in linking mitochondrial stress to epigenetic dysregulation. Potential strategies include enhancement of endogenous antioxidant defenses, modulation of NRF2–KEAP1 signaling, protection of glutathione redox balance, and reduction in excessive mitochondrial ROS [26,27,32]. Unlike nonspecific antioxidant supplementation, future redox-based approaches may need to focus on restoring physiological redox signaling rather than simply suppressing ROS production. This distinction is important because ROS also function as signaling molecules involved in stress adaptation, mitochondrial quality control, and transcriptional regulation. Excessive ROS neutralization could therefore impair adaptive responses, whereas insufficient control may permit sustained oxidative injury. Thus, redox-based therapy requires careful calibration of timing, dose, and cellular context.

Epigenetic modulators provide another attractive but challenging therapeutic direction. DNMTs, HDACs, histone acetyltransferases, and sirtuin-related pathways are potentially modifiable and may influence disease-associated transcriptional programs in the RPE. Preclinical studies suggest that HDAC inhibition can reduce retinal stress responses and partially restore gene-expression patterns consistent with chromatin relaxation [78]. However, direct epigenetic targeting raises major concerns regarding specificity and safety. Epigenetic enzymes regulate broad genomic programs across multiple retinal and non-retinal cell types, and non-selective modulation may produce off-target transcriptional effects, altered inflammatory responses, or disruption of normal cell identity. Therefore, epigenetic therapy for AMD should currently be viewed as a preclinical concept that requires improved isoform selectivity, cell-specific delivery, and clearer biomarkers of therapeutic response.

Delivery remains a central barrier to translating mitochondrial, antioxidant, or epigenetic strategies into ocular therapy. The RPE is protected by the outer blood–retina barrier, which limits systemic drug access. Intravitreal injection is clinically established but may not efficiently target the RPE, whereas subretinal delivery provides closer access but is more invasive and less suitable for repeated treatment [94,95]. Sustained-release systems, nanoparticles, lipid-based carriers, and viral or non-viral gene delivery platforms may improve retinal targeting, but each approach introduces additional concerns regarding biodistribution, immune activation, durability, dose control, and long-term safety [109,110,111]. For therapies intended to regulate epigenetic or mitochondrial pathways, delivery systems must ideally achieve sufficient RPE exposure while minimizing effects on photoreceptors, Müller glia, choroidal endothelial cells, and immune-active cells.

Therapeutic timing is likely to be equally important. In early AMD, when RPE dysfunction may still be partly reversible, interventions aimed at restoring mitochondrial function, redox buffering, metabolic homeostasis, and epigenetic flexibility may help delay progression. At this stage, antioxidant or mitochondrial-targeted strategies may be most rational because the goal is to preserve adaptive capacity before irreversible cell loss occurs. In advanced AMD, particularly geographic atrophy, loss of RPE and photoreceptors reduces the potential benefit of purely protective approaches. Treatment goals may therefore shift toward preserving remaining cells, limiting inflammatory amplification, supporting tissue repair, or combining metabolic protection with regenerative or cell-based strategies [32,92]. This stage-specific framework highlights the need for biomarkers that can identify therapeutic windows before structural degeneration becomes irreversible.

A translational pathway from mechanism to clinical application will require integration of molecular profiling, human-relevant models, and patient stratification. Multi-omics approaches combining transcriptomic, epigenomic, metabolomic, mitochondrial, and redox profiling may help identify regulatory nodes that are consistently altered across AMD stages [73,112]. Patient-derived iPSC-RPE, retinal organoids, advanced cybrid systems, and human donor tissue analyses may provide complementary platforms for testing whether mitochondrial or epigenetic interventions can reverse disease-relevant regulatory states. Longitudinal imaging and molecular biomarkers will also be essential to determine whether these interventions alter disease trajectory rather than only short-term cellular stress markers.

In summary, AMD should be considered a chronic degenerative disease shaped by interacting mitochondrial, metabolic, redox, inflammatory, and epigenetic disturbances rather than by a single initiating pathway. Epigenetic regulation provides an interface through which long-term stress exposure may become embedded into sustained transcriptional states in the RPE. However, direct manipulation of epigenetic enzymes remains limited by specificity, delivery, and safety concerns. By contrast, strategies that stabilize mitochondrial function, restore redox balance, and preserve metabolic resilience may indirectly maintain epigenetic flexibility and delay RPE decline. Future progress will depend on defining stage-specific therapeutic windows, developing RPE-targeted delivery systems, and validating intervention strategies in human-relevant AMD models.

## Figures and Tables

**Table 1:** Summary of key studies supporting epigenetic–mitochondrial–metabolic interactions in AMD.

Study/Evidence Type	Model or Sample	Mechanism Examined	Main Findings	Limitations	Relevance to AMD
Human epigenetic profiling	Blood and retinal samples from AMD patients	DNA methylation	Differential methylation patterns were identified in AMD, supporting the presence of systemic and ocular epigenetic alterations.	Cross-sectional design; tissue heterogeneity; limited causal inference.	High; provides direct AMD-associated epigenetic evidence.
Human donor RPE methylome	Postmortem RPE from AMD and control eyes	Genome-wide DNA methylation	Locus-specific methylation changes were reported near genes related to transcriptional regulation, extracellular matrix, and stress responses.	Donor variability; modest effect sizes; functional validation limited.	High; directly relevant to AMD-RPE.
Human donor ATAC-seq	Human AMD donor retina/RPE	Chromatin accessibility	AMD was associated with reduced chromatin accessibility, particularly in the RPE, suggesting altered regulatory architecture.	Endpoint tissue; causality and temporal sequence unclear.	High; supports chromatin remodeling in human AMD-RPE.
RPE oxidative stress models	Cultured RPE cells exposed to oxidative stress or cigarette smoke	ROS, autophagy, stress-response pathways	Oxidative stress altered survival, autophagy, and stress-response signaling in RPE cells.	In vitro conditions may not fully reproduce chronic AMD pathology.	Moderate to high; mechanistically relevant but model-dependent.
Mitochondrial dysfunction studies	Human AMD RPE and experimental models	Mitochondrial respiration, oxidative damage	Reduced mitochondrial function and increased oxidative injury were observed in AMD-relevant contexts.	Mechanistic directionality remains unresolved.	High; supports mitochondrial vulnerability in AMD.
Cybrid models	RPE cybrids carrying mitochondria from AMD donors	Mitonuclear signaling	AMD-associated mitochondrial backgrounds altered nuclear gene expression related to inflammation, oxidative stress, and epigenetic regulation.	Artificial system; lacks native retinal microenvironment.	Moderate; useful for mitochondria-to-nucleus signaling.
HDAC intervention models	Retinal degeneration or RPE stress models	Histone acetylation/HDAC activity	HDAC inhibition showed protective effects and partial restoration of stress-related gene expression.	Limited AMD-specific validation; off-target effects possible.	Moderate; supports therapeutic potential but requires caution.
miRNA studies	AMD-related ocular samples and RPE models	miRNA-mediated post-transcriptional regulation	Altered miRNA profiles were linked to apoptosis, inflammation, mitochondrial regulation, and oxidative stress.	Variable reproducibility; many findings are associative.	Moderate; supports RNA-level modulation of RPE stress responses.
lncRNA studies	Ocular samples and experimental AMD-related models	lncRNA-mediated transcriptional or chromatin regulation	lncRNA alterations were associated with inflammatory and stress-response pathways.	Functional validation limited; high context dependence.	Low to moderate; emerging but not yet definitive.
m6A-related evidence	Retina/RPE-relevant or extrapolated stress models	RNA modification and stress-responsive translation	m6A may regulate mRNA stability and translation under metabolic or oxidative stress.	Direct AMD-RPE evidence remains limited.	Low to moderate; conceptually relevant but largely inferred.

## Data Availability

No new data were created or analyzed in this study. Data sharing is not applicable to this article.

## Abbreviations

The following abbreviations are used in this manuscript:

α-KG	α-Ketoglutarate
AMD	Age-related macular degeneration
APOE	Apolipoprotein E
ATAC-seq	Assay for transposase-accessible chromatin with sequencing
ATP	Adenosine triphosphate
ChIP-seq	Chromatin immunoprecipitation sequencing
CUT&Tag	Cleavage under targets and tagmentation
DNA	Deoxyribonucleic acid
DNMTs	DNA methyltransferases
ECM	Extracellular matrix
ELOVL2	Elongation of very long-chain fatty acids protein 2
H3K27ac	Histone H3 lysine 27 acetylation
H3K9me3	Histone H3 lysine 9 trimethylation
HDAC	Histone deacetylase
iPSC-RPE	Induced pluripotent stem cell-derived retinal pigment epithelium
KEAP1	Kelch-like ECH-associated protein 1
lncRNA	long non-coding RNA
m6A	N6-methyladenosine
METTL3	Methyltransferase-like 3
miRNA	microRNA
NAD^+^	Nicotinamide adenine dinucleotide
NRF2	Nuclear factor erythroid 2-related factor 2
PARP	Poly(ADP-ribose) polymerase
RNA	Ribonucleic acid
ROS	Reactive oxygen species
RPE	Retinal pigment epithelium
SAM	S-adenosylmethionine
SIRT1	Sirtuin 1
SIRT3	Sirtuin 3
TET	Ten-eleven translocation
VEGF	Vascular endothelial growth factor
WTAP	Wilms tumor 1-associated protein
YTHDC1	YTH domain-containing protein 1
YTHDF1	YTH N6-methyladenosine RNA binding protein 1

## References

[1] Zou M., Zhang Y., Chen A., Young C.A., Li Y., Zheng D., Jin G. (2021). Variations and trends in global disease burden of age-related macular degeneration: 1990–2017. Acta Ophthalmol..

[2] Wong W.L., Su X., Li X., Cheung C.M., Klein R., Cheng C.Y., Wong T.Y. (2014). Global prevalence of age-related macular degeneration and disease burden projection for 2020 and 2040: A systematic review and meta-analysis. Lancet Glob. Health.

[3] Liu M.M., Chan C.C., Tuo J. (2012). Genetic mechanisms and age-related macular degeneration: Common variants, rare variants, copy number variations, epigenetics, and mitochondrial genetics. Hum. Genom..

[4] Ambati J., Fowler B.J. (2012). Mechanisms of age-related macular degeneration. Neuron.

[5] Fritsche L.G., Igl W., Bailey J.N., Grassmann F., Sengupta S., Bragg-Gresham J.L., Burdon K.P., Hebbring S.J., Wen C., Gorski M. (2016). A large genome-wide association study of age-related macular degeneration highlights contributions of rare and common variants. Nat. Genet..

[6] Grassmann F., Kiel C., Zimmermann M.E., Gorski M., Grassmann V., Stark K., Heid I.M., Weber B.H. (2017). Genetic pleiotropy between age-related macular degeneration and 16 complex diseases and traits. Genome Med..

[7] Corso-Díaz X., Jaeger C., Chaitankar V., Swaroop A. (2018). Epigenetic control of gene regulation during development and disease: A view from the retina. Prog. Retin. Eye Res..

[8] Moore S.M., Christoforidis J.B. (2023). Advances in Ophthalmic Epigenetics and Implications for Epigenetic Therapies: A Review. Genes.

[9] López-Otín C., Blasco M.A., Partridge L., Serrano M., Kroemer G. (2013). The hallmarks of aging. Cell.

[10] Pal S., Tyler J.K. (2016). Epigenetics and aging. Sci. Adv..

[11] Kenney M.C., Nashine S. (2020). Further understanding of epigenetic dysfunction of the retinal pigment epithelium in AMD. Expert Rev. Ophthalmol..

[12] Wang J., Zibetti C., Shang P., Sripathi S.R., Zhang P., Cano M., Hoang T., Xia S., Ji H., Merbs S.L. (2018). ATAC-Seq analysis reveals a widespread decrease of chromatin accessibility in age-related macular degeneration. Nat. Commun..

[13] Sparrow J.R., Hicks D., Hamel C.P. (2010). The retinal pigment epithelium in health and disease. Curr. Mol. Med..

[14] Caceres P.S., Rodriguez-Boulan E. (2020). Retinal pigment epithelium polarity in health and blinding diseases. Curr. Opin. Cell Biol..

[15] Strauss O. (2005). The retinal pigment epithelium in visual function. Physiol. Rev..

[16] Kim J., Lee Y.J., Won J.Y. (2021). Molecular Mechanisms of Retinal Pigment Epithelium Dysfunction in Age-Related Macular Degeneration. Int. J. Mol. Sci..

[17] Keeling E., Lotery A.J., Tumbarello D.A., Ratnayaka J.A. (2018). Impaired Cargo Clearance in the Retinal Pigment Epithelium (RPE) Underlies Irreversible Blinding Diseases. Cells.

[18] Ferrington D.A., Fisher C.R., Kowluru R.A. (2020). Mitochondrial Defects Drive Degenerative Retinal Diseases. Trends Mol. Med..

[19] Kaarniranta K., Uusitalo H., Blasiak J., Felszeghy S., Kannan R., Kauppinen A., Salminen A., Sinha D., Ferrington D. (2020). Mechanisms of mitochondrial dysfunction and their impact on age-related macular degeneration. Prog. Retin. Eye Res..

[20] Terluk M.R., Kapphahn R.J., Soukup L.M., Gong H., Gallardo C., Montezuma S.R., Ferrington D.A. (2015). Investigating mitochondria as a target for treating age-related macular degeneration. J. Neurosci..

[21] Keeling E., Chatelet D.S., Tan N.Y.T., Khan F., Richards R., Thisainathan T., Goggin P., Page A., Tumbarello D.A., Lotery A.J. (2020). 3D-Reconstructed Retinal Pigment Epithelial Cells Provide Insights into the Anatomy of the Outer Retina. Int. J. Mol. Sci..

[22] Ratnayaka J.A., Keeling E. (2022). Serial block face scanning electron microscopy reveals novel organizational details of the retinal pigment epithelium. Neural Regen. Res..

[23] Kaštelan S., Antunica A.G., Konjevoda S., Tomić Z., Sarić A., Kulaš M., Kulaš L., Begović E.K., Čanović S., Kovačević P. (2026). Mitochondrial ROS in Retinal Neurodegeneration: Thresholds, Quality Control Failure, and Precision Therapeutic Windows. Biomolecules.

[24] Gurubaran I.S. (2024). Mitochondrial damage and clearance in retinal pigment epithelial cells. Acta Ophthalmol..

[25] Ngo V., Duennwald M.L. (2022). Nrf2 and Oxidative Stress: A General Overview of Mechanisms and Implications in Human Disease. Antioxidants.

[26] Zhang L., Xu L.Y., Tang F., Liu D., Zhao X.L., Zhang J.N., Xia J., Wu J.J., Yang Y., Peng C. (2024). New perspectives on the therapeutic potential of quercetin in non-communicable diseases: Targeting Nrf2 to counteract oxidative stress and inflammation. J. Pharm. Anal..

[27] Duan W., Li T., Zhang Y., Sun B., Liu R.H. (2026). Microbiota-Derived Metabolites Associated with Oats and Bran Attenuate Inflammation and Oxidative Stress via the Keap1-Nrf2 Pathway in Zebrafish. Nutrients.

[28] Sailis A.B. (2026). NRF2-KEAP1 as a Redox Signal-Resolution Circuit: Beyond the Antioxidant Switch. Prog. Biophys. Mol. Biol..

[29] Zhang S., Duan S., Xie Z., Bao W., Xu B., Yang W., Zhou L. (2022). Epigenetic Therapeutics Targeting NRF2/KEAP1 Signaling in Cancer Oxidative Stress. Front. Pharmacol..

[30] Crisman E., Duarte P., Dauden E., Cuadrado A., Rodríguez-Franco M.I., López M.G., León R. (2023). KEAP1-NRF2 protein-protein interaction inhibitors: Design, pharmacological properties and therapeutic potential. Med. Res. Rev..

[31] Wu J., Liu L.L., Cao M., Hu A., Hu D., Luo Y., Wang H., Zhong J.N. (2021). DNA methylation plays important roles in retinal development and diseases. Exp. Eye Res..

[32] Datta S., Cano M., Ebrahimi K., Wang L., Handa J.T. (2017). The impact of oxidative stress and inflammation on RPE degeneration in non-neovascular AMD. Prog. Retin. Eye Res..

[33] Lenin R.R., Koh Y.H., Zhang Z., Yeo Y.Z., Parikh B.H., Seah I., Wong W., Su X. (2023). Dysfunctional Autophagy, Proteostasis, and Mitochondria as a Prelude to Age-Related Macular Degeneration. Int. J. Mol. Sci..

[34] Ong J., Selvam A., Driban M., Zarnegar A., Morgado Mendes Antunes Da Silva S.I., Joy J., Rossi E.A., Vande Geest J.P., Sahel J.A., Chhablani J. (2025). Characterizing Bruch’s membrane: State-of-the-art imaging, computational segmentation, and biologic models in retinal disease and health. Prog. Retin. Eye Res..

[35] Clark S.J., Perveen R., Hakobyan S., Morgan B.P., Sim R.B., Bishop P.N., Day A.J. (2010). Impaired binding of the age-related macular degeneration-associated complement factor H 402H allotype to Bruch’s membrane in human retina. J. Biol. Chem..

[36] Nava M.M., Miroshnikova Y.A., Biggs L.C., Whitefield D.B., Metge F., Boucas J., Vihinen H., Jokitalo E., Li X., García Arcos J.M. (2020). Heterochromatin-Driven Nuclear Softening Protects the Genome against Mechanical Stress-Induced Damage. Cell.

[37] Curcio C.A., Johnson M., Rudolf M., Huang J.D. (2011). The oil spill in ageing Bruch membrane. Br. J. Ophthalmol..

[38] Curcio C.A., Johnson M., Huang J.D., Rudolf M. (2009). Aging, age-related macular degeneration, and the response-to-retention of apolipoprotein B-containing lipoproteins. Prog. Retin. Eye Res..

[39] Scheepers R., Pettet G.J., van Heijster P., Araujo R.P. (2020). Cholesterol Regulation in Age-Related Macular Degeneration: A Framework for Mathematical Modelling of Drusen Biogenesis. Bull. Math. Biol..

[40] Chu Y.K., Lee S.C., Byeon S.H. (2013). VEGF rescues cigarette smoking-induced human RPE cell death by increasing autophagic flux: Implications of the role of autophagy in advanced age-related macular degeneration. Investig. Ophthalmol. Vis. Sci..

[41] Handa J.T., Bowes Rickman C., Dick A.D., Gorin M.B., Miller J.W., Toth C.A., Ueffing M., Zarbin M., Farrer L.A. (2019). A systems biology approach towards understanding and treating non-neovascular age-related macular degeneration. Nat. Commun..

[42] Oliver V.F., Jaffe A.E., Song J., Wang G., Zhang P., Branham K.E., Swaroop A., Eberhart C.G., Zack D.J., Qian J. (2015). Differential DNA methylation identified in the blood and retina of AMD patients. Epigenetics.

[43] Porter L.F., Saptarshi N., Fang Y., Rathi S., den Hollander A.I., de Jong E.K., Clark S.J., Bishop P.N., Olsen T.W., Liloglou T. (2019). Whole-genome methylation profiling of the retinal pigment epithelium of individuals with age-related macular degeneration reveals differential methylation of the SKI, GTF2H4, and TNXB genes. Clin. Epigenet..

[44] He Z., Zhang R., Jiang F., Hou W., Hu C. (2018). Role of genetic and environmental factors in DNA methylation of lipid metabolism. Genes Dis..

[45] Mentch S.J., Mehrmohamadi M., Huang L., Liu X., Gupta D., Mattocks D., Gómez Padilla P., Ables G., Bamman M.M., Thalacker-Mercer A.E. (2015). Histone Methylation Dynamics and Gene Regulation Occur through the Sensing of One-Carbon Metabolism. Cell Metab..

[46] Gemenetzi M., Lotery A.J. (2014). The role of epigenetics in age-related macular degeneration. Eye.

[47] Caputo V., Strafella C., Termine A., Fabrizio C., Ruffo P., Cusumano A., Giardina E., Ricci F., Cascella R. (2021). Epigenomic signatures in age-related macular degeneration: Focus on their role as disease modifiers and therapeutic targets. Eur. J. Ophthalmol..

[48] Kumar A., Choudhary A., Munshi A. (2024). Epigenetic reprogramming of mtDNA and its etiology in mitochondrial diseases. J. Physiol. Biochem..

[49] Joshi K., Liu S., Breslin S.J.P., Zhang J. (2022). Mechanisms that regulate the activities of TET proteins. Cell. Mol. Life Sci..

[50] Guo Y., Yu S., Zhang C., Kong A.N. (2015). Epigenetic regulation of Keap1-Nrf2 signaling. Free Radic. Biol. Med..

[51] Ruzycki P.A., Tran N.M., Kefalov V.J., Kolesnikov A.V., Chen S. (2015). Graded gene expression changes determine phenotype severity in mouse models of CRX-associated retinopathies. Genome Biol..

[52] Field A.E., Robertson N.A., Wang T., Havas A., Ideker T., Adams P.D. (2018). DNA Methylation Clocks in Aging: Categories, Causes, and Consequences. Mol. Cell.

[53] Li X., Wang J., Wang L., Gao Y., Feng G., Li G., Zou J., Yu M., Li Y.F., Liu C. (2022). Lipid metabolism dysfunction induced by age-dependent DNA methylation accelerates aging. Signal Transduct. Target. Ther..

[54] Gao F., Tom E., Rydz C., Cho W., Kolesnikov A.V., Sha Y., Papadam A., Jafari S., Joseph A., Ahanchi A. (2025). Retinal polyunsaturated fatty acid supplementation reverses aging-related vision decline in mice. Sci. Transl. Med..

[55] Hansman D.S., Du J., Casson R.J., Peet D.J. (2025). Eye on the horizon: The metabolic landscape of the RPE in aging and disease. Prog. Retin. Eye Res..

[56] Stuard Sambhariya W., Bowes Rickman C., D’Amore P.A., Corradetti G., Hageman G.S., Howell G.R., Marola O.J., Phatnani H., Philp N.J., Sinha D. (2026). Age-related macular degeneration and cerebral amyloid angiopathy have similar pathologies from cholesterol-APOE-amyloid-β-complement mediated inflammation. Prog. Retin. Eye Res..

[57] Lyssenko N.N., Haider N., Picataggi A., Cipollari E., Jiao W., Phillips M.C., Rader D.J., Chavali V.R.M. (2018). Directional ABCA1-mediated cholesterol efflux and apoB-lipoprotein secretion in the retinal pigment epithelium. J. Lipid Res..

[58] Gabrielle P.H. (2022). Lipid metabolism and retinal diseases. Acta Ophthalmol..

[59] Delrue C., Speeckaert R., Speeckaert M.M. (2026). Molecular Crosstalk in Age-Related Macular Degeneration: Integrating Oxidative Stress, Inflammation, microRNAs, and Genetic Susceptibility Toward Precision Therapeutics. Biomolecules.

[60] Dang S., Jain A., Dhanda G., Bhattacharya N., Bhattacharya A., Senapati S. (2024). One carbon metabolism and its implication in health and immune functions. Cell Biochem. Funct..

[61] Majumder A., Bano S., Nayak K.B. (2024). The Pivotal Role of One-Carbon Metabolism in Neoplastic Progression During the Aging Process. Biomolecules.

[62] Moore L.D., Le T., Fan G. (2013). DNA methylation and its basic function. Neuropsychopharmacology.

[63] Kaelin W.G., McKnight S.L. (2013). Influence of metabolism on epigenetics and disease. Cell.

[64] Breitling L.P., Yang R., Korn B., Burwinkel B., Brenner H. (2011). Tobacco-smoking-related differential DNA methylation: 27K discovery and replication. Am. J. Hum. Genet..

[65] Zhang S.M., Fan B., Li Y.L., Zuo Z.Y., Li G.Y. (2023). Oxidative Stress-Involved Mitophagy of Retinal Pigment Epithelium and Retinal Degenerative Diseases. Cell. Mol. Neurobiol..

[66] Bannister A.J., Kouzarides T. (2011). Regulation of chromatin by histone modifications. Cell Res..

[67] Gunes S., Hekim N., Ergun S., Alkan E.N., Can C. (2025). Histone and Non-histone Reversible Acetylation in Development, Aging, and Disease. Results Probl. Cell Differ..

[68] Voigt A.P., Mulfaul K., Mullin N.K., Flamme-Wiese M.J., Giacalone J.C., Stone E.M., Tucker B.A., Scheetz T.E., Mullins R.F. (2019). Single-cell transcriptomics of the human retinal pigment epithelium and choroid in health and macular degeneration. Proc. Natl. Acad. Sci. USA.

[69] Huang Z., Cai L., Tu B.P. (2015). Dietary control of chromatin. Curr. Opin. Cell Biol..

[70] Morrison A.J. (2020). Chromatin-remodeling links metabolic signaling to gene expression. Mol. Metab..

[71] Policarpi C., Munafò M., Tsagkris S., Carlini V., Hackett J.A. (2024). Systematic epigenome editing captures the context-dependent instructive function of chromatin modifications. Nat. Genet..

[72] Zhao Y., Garcia B.A. (2015). Comprehensive Catalog of Currently Documented Histone Modifications. Cold Spring Harb. Perspect. Biol..

[73] Liao M., Zhu X., Lu Y., Yi X., Hu Y., Zhao Y., Ye Z., Guo X., Liang M., Jin X. (2024). Multi-omics profiling of retinal pigment epithelium reveals enhancer-driven activation of RANK-NFATc1 signaling in traumatic proliferative vitreoretinopathy. Nat. Commun..

[74] Husain S., Obert E., Singh S., Schnabolk G. (2024). Inhibition of HDAC1 and 3 in the Presence of Systemic Inflammation Reduces Retinal Degeneration in a Model of Dry Age-Related Macular Degeneration. J. Ocul. Pharmacol. Ther..

[75] Lugassy Y., Berent E., Tarony L., Jeries S., Ziv T., Savion N., Eldar-Finkelman H. (2025). HDAC inhibition protects RPE cells from oxidative stress via enhanced mitochondrial fusion, cytoskeletal repair, and Nrf-2 activation. Free Radic. Biol. Med..

[76] Ozawa Y., Kubota S., Narimatsu T., Yuki K., Koto T., Sasaki M., Tsubota K. (2010). Retinal aging and sirtuins. Ophthalmic Res..

[77] Nashine S., Nesburn A.B., Kuppermann B.D., Kenney M.C. (2019). Age-related macular degeneration (AMD) mitochondria modulate epigenetic mechanisms in retinal pigment epithelial cells. Exp. Eye Res..

[78] Luo T., Li C., Zhou L., Sun H., Yang M.M. (2025). Protein Acetylation in Age-Related Macular Degeneration: Mechanisms, Roles, and Therapeutic Perspectives. Investig. Ophthalmol. Vis. Sci..

[79] Li Y., Seto E. (2016). HDACs and HDAC Inhibitors in Cancer Development and Therapy. Cold Spring Harb. Perspect. Med..

[80] Picard M., Wallace D.C., Burelle Y. (2016). The rise of mitochondria in medicine. Mitochondrion.

[81] Zong W., Gong Y., Sun W., Li T., Wang Z.Q. (2022). PARP1: Liaison of Chromatin Remodeling and Transcription. Cancers.

[82] Mazzeo L., Arsenijevic Y., Berger A. (2025). Exploring Histone Modifications in Inherited Retinal Disorders. Adv. Exp. Med. Biol..

[83] Chandel N.S. (2014). Mitochondria as signaling organelles. BMC Biol..

[84] Quirós P.M., Mottis A., Auwerx J. (2016). Mitonuclear communication in homeostasis and stress. Nat. Rev. Mol. Cell Biol..

[85] Zhang B., Pan C., Feng C., Yan C., Yu Y., Chen Z., Guo C., Wang X. (2022). Role of mitochondrial reactive oxygen species in homeostasis regulation. Redox Rep..

[86] Panvini A.R., Gvritishvili A., Galvan H., Nashine S., Atilano S.R., Kenney M.C., Tombran-Tink J. (2022). Differential mitochondrial and cellular responses between H vs. J mtDNA haplogroup-containing human RPE transmitochondrial cybrid cells. Exp. Eye Res..

[87] Salimiaghdam N., Singh L., Singh M.K., Chwa M., Atilano S.R., Mohtashami Z., Nesburn A.B., Kuppermann B.D., Lu S.Y., Kenney M.C. (2022). Impacts of Bacteriostatic and Bactericidal Antibiotics on the Mitochondria of the Age-Related Macular Degeneration Cybrid Cell Lines. Biomolecules.

[88] Rahman J., Rahman S. (2018). Mitochondrial medicine in the omics era. Lancet.

[89] Léveillard T., Philp N.J., Sennlaub F. (2019). Is Retinal Metabolic Dysfunction at the Center of the Pathogenesis of Age-related Macular Degeneration?. Int. J. Mol. Sci..

[90] Yu J.J., Azzam D.B., Chwa M., Schneider K., Cho J.H., Hsiang C., Klassen H., Kenney M.C., Yang J. (2021). Age-Related Macular Degeneration (AMD) Transmitochondrial Cybrids Protected from Cellular Damage and Death by Human Retinal Progenitor Cells (hRPCs). Stem Cells Int..

[91] Zhang M., Jiang N., Chu Y., Postnikova O., Varghese R., Horvath A., Cheema A.K., Golestaneh N. (2020). Dysregulated metabolic pathways in age-related macular degeneration. Sci. Rep..

[92] Hyttinen J., Blasiak J., Tavi P., Kaarniranta K. (2021). Therapeutic potential of PGC-1α in age-related macular degeneration (AMD)—The involvement of mitochondrial quality control, autophagy, and antioxidant response. Expert Opin. Ther. Targets.

[93] Hyttinen J.M.T., Blasiak J., Felszeghy S., Kaarniranta K. (2021). MicroRNAs in the regulation of autophagy and their possible use in age-related macular degeneration therapy. Ageing Res. Rev..

[94] Berber P., Grassmann F., Kiel C., Weber B.H. (2017). An Eye on Age-Related Macular Degeneration: The Role of MicroRNAs in Disease Pathology. Mol. Diagn. Ther..

[95] Du S.W., Palczewski K. (2022). MicroRNA regulation of critical retinal pigment epithelial functions. Trends Neurosci..

[96] Yang Y.J., Wang Y., Deng Y., Liu X.Q., Lu J., Peng J., Li J., Zhou Y.S., Zhu H.A., Li B. (2023). Lycium barbarum Polysaccharides Regulating miR-181/Bcl-2 Decreased Autophagy of Retinal Pigment Epithelium with Oxidative Stress. Oxidative Med. Cell. Longev..

[97] Komatsu S., Kitai H., Suzuki H.I. (2023). Network Regulation of microRNA Biogenesis and Target Interaction. Cells.

[98] ElShelmani H., Brennan I., Kelly D.J., Keegan D. (2021). Differential Circulating MicroRNA Expression in Age-Related Macular Degeneration. Int. J. Mol. Sci..

[99] Natoli R., Fernando N. (2018). MicroRNA as Therapeutics for Age-Related Macular Degeneration. Adv. Exp. Med. Biol..

[100] Wang H., Wang C., Yao Y., Duan J., Liang Y., Shang Q. (2023). Analysis of long noncoding RNAs in the aqueous humor of wet age-related macular degeneration. Exp. Eye Res..

[101] Wawrzyniak O., Zarębska Ż., Rolle K., Gotz-Więckowska A. (2018). Circular and long non-coding RNAs and their role in ophthalmologic diseases. Acta Biochim. Pol..

[102] Statello L., Guo C.J., Chen L.L., Huarte M. (2021). Gene regulation by long non-coding RNAs and its biological functions. Nat. Rev. Mol. Cell Biol..

[103] Menon M., Mohammadi S., Davila-Velderrain J., Goods B.A., Cadwell T.D., Xing Y., Stemmer-Rachamimov A., Shalek A.K., Love J.C., Kellis M. (2019). Single-cell transcriptomic atlas of the human retina identifies cell types associated with age-related macular degeneration. Nat. Commun..

[104] Zaccara S., Ries R.J., Jaffrey S.R. (2019). Reading, writing and erasing mRNA methylation. Nat. Rev. Mol. Cell Biol..

[105] He L., Li H., Wu A., Peng Y., Shu G., Yin G. (2019). Functions of N6-methyladenosine and its role in cancer. Mol. Cancer.

[106] Ponzetti M., Rucci N., Falone S. (2023). RNA methylation and cellular response to oxidative stress-promoting anticancer agents. Cell Cycle.

[107] Kovács-Öller T., Völgyi B. (2023). Molecular Mechanisms of Retinal Degeneration and How to Avoid It. Int. J. Mol. Sci..

[108] Tan W., Zou J., Yoshida S., Jiang B., Zhou Y. (2020). The Role of Inflammation in Age-Related Macular Degeneration. Int. J. Biol. Sci..

[109] Mehta N.J., Mehta S.N. (2024). Nanotechnology in Retinal Disease: Current Concepts and Future Directions. J. Ocul. Pharmacol. Ther..

[110] Siontas O., Brown M., Ahn S. (2025). Toward improved AAV gene therapies for retinal disorders: Challenges and advances. Regen. Med..

[111] Lin X., Zhou Y., Lv K., Wu W., Chen C. (2025). Nanomedicine-Based Ophthalmic Drug Delivery Systems for the Treatment of Ocular Diseases. Int. J. Nanomed..

[112] Orozco L.D., Chen H.H., Cox C., Katschke K.J., Arceo R., Espiritu C., Caplazi P., Nghiem S.S., Chen Y.J., Modrusan Z. (2020). Integration of eQTL and a Single-Cell Atlas in the Human Eye Identifies Causal Genes for Age-Related Macular Degeneration. Cell Rep..

